# *Pdgfrb* is a direct regulatory target of TGFβ signaling in atrioventricular cushion mesenchymal cells

**DOI:** 10.1371/journal.pone.0175791

**Published:** 2017-04-20

**Authors:** Yin Peng, Shun Yan, Dongquan Chen, Xiangqin Cui, Kai Jiao

**Affiliations:** 1 Division of Research, Department of Genetics, The University of Alabama at Birmingham, Birmingham, Alabama, United States of America; 2 Division of Preventive Medicine, Department of Medicine, The University of Alabama at Birmingham, Birmingham, Alabama, United States of America; 3 Department of Biostatistics, The University of Alabama at Birmingham, Birmingham, Alabama, United States of America; University of South Alabama Mitchell Cancer Institute, UNITED STATES

## Abstract

Cushion formation is the initial step for the development of valvuloseptal structures in mammalian hearts. TGFβ signaling plays critical roles in multiple steps of cushion morphogenesis. We used a newly developed conditional immortal atrioventricular cushion mesenchymal cell line, tsA58-AVM, to identify the TGFβ regulatory target genes through microarray analysis. Expression of ~1350 genes was significantly altered by TGFβ1 treatment. Subsequent bioinformatic analysis of TGFβ activated genes revealed that PDGF-BB signaling is the top hit as the potential upstream regulator. Among the 37 target molecules, 10 genes known to be involved in valve development and hemostasis were selected for quantitative reverse transcription polymerase chain reaction (qRT-PCR) analysis. Our results confirmed that they are all upregulated by TGFβ1 stimulation in tsA58-AVM cells and in primary atrioventricular cushion cells. We focused on examining regulation of *Pdgfrb* by TGFβ1, which encodes a tyrosine kinase receptor for PDGF-BB. We found that the ~150bp *Pdgfrb* promoter can respond to TGFβ stimulation and that this response relies on the two SP1 binding sites within the promoter. Co-immunoprecipitation analysis confirmed SP1 interacts with SMAD2 in a TGFβ-dependent fashion. Furthermore, SMAD2 is associated with the *Pdgfrb* promoter and this association is diminished by knocking down expression of *Sp1*. Our data therefore collectively suggest that upon TGFβ stimulation, SP1 recruits SMAD2 to the promoter of *Pdgfrb* to up-regulate its expression and thus *Pdgfrb* is a direct downstream target of the TGFβ/SMAD2 signaling.

## Introduction

Normal development of valvuloseptal structures is essential for a mammalian heart to be properly partitioned into four chambers. Up to 30% of congenital heart defects are caused by malformation of valves [1]. Valvulogenesis in mice is initiated with cushion formation in the atrioventricular (AV) canal region at E9.0 and the outflow tract region at E10.0. Shortly after, a group of endocardial cells in the AV cushion and OFT conal cushion undergo epithelial-mesenchyme-transition (EMT) to become cushion mesenchymal cells [1–12]. These cellularized cushions serve as the primordia of valves and septa to ensure unidirectional blood flow in embryos. At later developmental stages, cushions go through complicated remodeling processes to mature into the final valve and septum structures.

Transforming Growth Factor beta (TGFβ) signaling plays critical roles in many biological/pathological processes, including development of valvuloseptal structures. TGFβ signaling is initiated when homo-dimers of ligands (including TGFβ1, 2 and 3) bind to and bring together the type I and II receptors at cell membranes. The type II receptor phosphorylates (activates) the type I receptor, which subsequently phosphorylates SMAD2 and SMAD3, which are also known as TGFβ Receptor-activated SMADs (R-SMADs). Phosphorylated R-SMADs associate with SMAD4 (co-SMAD) and translocate to the nucleus to regulate transcription of target genes [13–18]. SMAD3 and SMAD4 can directly bind to DNA target sites, called SMAD-Binding Elements (SBEs) [19, 20]. Unlike SMAD3, SMAD2 does not directly interact with SBEs; SMAD2 can be loaded to DNA through interaction with other sequence-specific transcription factors to modulate gene expression [18, 21].

The functions of TGFβ signaling in regulating cushion development in the AV canal region have been well documented. In *ex vivo* collagen gel analyses, TGFβ ligands can substitute for the overlying myocardium to activate EMT [22–24]. Inhibition of TGFβ signaling with an antisense oligonucleotide against *TGFβ3* mRNA or with neutralizing antiserums against TGFβ ligands, receptors, or co-receptors blocks EMT [25–28]. *Tgfb2*^*-/-*^ mice display complex heart defects, including double-outlet-right-ventricle, atrial septal defect, ventricular septal defect, an overriding tricuspid valve and failure in myocardialization [29, 30]. The overriding of tricuspid valve observed in 25% of *Tgfb2*^*-/-*^ mice conclusively demonstrated that TGFβ signaling is required for normal AV valve development. A later study further showed that *Tgfb2* is required for normal cushion mesenchymal cell differentiation [31]. *TGFβ1*^*-/-*^ [32] and *TGFβ3*^*-/-*^ [33] mice do not display obvious valvular defects. The discrepancy between *in vivo* mouse studies and *ex vivo* explant assays are likely due to complementation of by the remaining TGFβ ligands present in mice. Our previous study showed that endothelial/endocardial inactivation of *Tgfbr2* leads to a double-inlet-left-ventricle defect, which is at least partially due to abnormal cell proliferation in AV cushion mesenchymal cells [34]. Endothelial inactivation of *Acvr1* (*Alk2*), which encodes a type I receptor capable of mediating both TGFβ and Bone Morphogenetic Protein (BMP) signaling, significantly reduced cushion mesenchymal cell formation in the AV canal region [35]. A similar hypocellular AV cushion defect was observed in mice with *Tgfbr1* (Alk5) deleted in the endothelial cells [36]. Evaluation of the role of SMAD proteins in valve development has primarily focused on SMAD4. Endothelial deletion of *Smad4* led to hypocellular AV cushions [37, 38]. Since SMAD4 is a co-SMAD acting with both TGFβ- and BMP- activated R-SMADs, the observed AV defects can be potentially due to the combined effect of impaired TGFβ and BMP activities.

Compared to the myocardial cells in mouse embryonic hearts, the number of AV cushion mesenchymal cells is greatly limited. To facility large scale molecular and cellular analyses on these types of cells, we developed a conditional immortal AV cushion mesenchymal cell line, tsA58-AVM [39]. These cells express temperature-sensitive mutant large T antigen under the control of a γ-interferon inducible promoter. They can be maintained and stored like immortal cells under the permissive condition (at 33°C with γ-interferon), while under the restrictive condition (at 37°C without γ-interferon), they behave like primary cultured cells. In this study, we used tsA58-AVM cells to identify TGFβ regulatory target genes through microarray analysis. Our bioinformatic analysis revealed that platelet-derived growth factor (PDGF)-BB is the top upstream regulator of TGFβ-activated genes in tsA58-AVM cells and our combined functional tests identified *Pdgfrb* as a direct target of the TGFβ/SMAD2 signaling cascade.

PDGF-BB refers to the PDGF-B homodimer cytokine, whose activity can be mediated through PDGFRA and PDGFRB tyrosine kinase receptors [40, 41]. Inactivation of *Pdgf-B* or *Pdgfrb* in mice led to complex cardiac defects, including underdeveloped AV valves, impaired development of coronary arteries, abnormal alignment of the outflow tract, and hypoplastic ventricles [42–45]. Functional interaction between PDGF signaling and TGFβ/SMAD signaling has been well documented in various cell types (*e*.*g*. [46–48]). Our study represents the first, to our knowledge, in which a gene encoding a receptor of PDGF, *Pdgfrb*, is shown to be directly regulated by TGFβ/SMAD2 signaling.

## Materials and methods

### Cell culture, DNA transfection and co-immunoprecipitation (co-IP)

Derivation and characterization of tsA58-AVM cells from AV cushion mesenchymal cells were described previously [39]. These cells were maintained in DMEM with 10% Fetal Bovine Serum (FBS) and 5 units/ml γ-interferon (Peprotech) at 33°C. Before experiments, cells were shifted to the restrictive condition (37°C without γ-interferon) for three days to allow the large T antigen to be degraded. Cells were used between 4 and 14 days after being shifted to the restrictive condition. Transfection of DNA and short-inhibitory RNAs (siRNAs) was achieved through electroporation using the Neon transfection system (Invitrogen), as described previously [39]. Electroporation was performed with 1300 volts for 20ms, two pulses. The pre-made Dicer-Substrate siRNAs (DsiRNAs) against mouse *Sp1*, including mm.Ri.Sp1.13.1 and mm.Ri.Sp1.13.2, and a scrambled DsiRNA were purchased from IDT. The mm.Ri.Sp1.13.1 and mm.Ri.Sp1.13.2 DsiRNAs target bp7562-7586 and bp722-746 of *Sp1* cDNA, respectively. For co-IP analyses, tsA58-AVM cells growing under the restrictive condition were treated with 5ng/ml TGFβ1 or BSA for 30 minutes and lysed with lysis buffer (50mM Tris-HCl, pH 7.4, 0.1% Triton, 0.1% NP40, 0.1% Tween-20, 5mM EDTA, 10% glycerol, 1 x proteinase inhibitor from Roche). The lysate was incubated with a SP1 antibody (1/50, Abcam, ab13370) overnight at 4°C. The next day Protein A/G beads were added to the samples. After a four hour incubation, the beads were spun down and washed three times with lysis buffer. Proteins were then eluted using Laemmli buffer followed by Western analysis using an antibody against SP1 (1/1000) or SMAD2 (1/1000, Cell Signaling, #5339).

### Microarray and quantitative reverse transcription-polymerase chain reaction (qRT-PCR) analyses

tsA58-AVM cells grown under the restrictive condition were treated with 5ng/ml TGFβ1 (R&D) or BSA for eight hours. Total RNA was isolated from the cells using the RNeasy-Plus kit (Qiagen). Microarray analysis was performed using the Affymetrix GeneChip-Mouse-Genome-420-2.0 Array by the UAB Heflin Genomic Core Facility, as described in our previous studies [39, 49]. Data were averaged from three independent biological samples for each condition. Isolation of primary AV cushion mesenchymal cells was performed as described in [50]. Briefly, AV canal tissues from E9.5 mouse embryos were isolated, cut to open longitudinally and placed onto hydrated type I collagen gels with the endocardial layer facing down. After 12 hour incubation at 37°C in a cell culture incubator, the muscle layer of the AV explants was carefully removed and the remaining cells were then treated with 5ng/ml TGFβ1 (R&D) or BSA for six hours. Total RNA was isolated from these cells using Arcturus PicoPure kit (Life Technologies). RNA signals were linearly amplified using the Illumina RNA amplification kit (Life Technologies). For qRT-PCR analyses, RNA samples acquired from tsAV58-AVM cells or primary AV cushion cells were reversely transcribed using SuperScript™ III First-Strand Synthesis System (Invitrogen, #18080051) followed by PCR using LightCycler® 480 SYBR Green I Master (Roche, #4707516001). qPCR was performed on the Roche LightCycler480 Real Time PCR machine. *Hprt* was used as the loading control for all qRT-PCR studies. The relative amount of PCR product was calculated using the ΔΔCt method, as performed previously [51]. The qRT-PCR primers are listed in S1 Table. *Hprt* was used as the loading control for all qRT-PCR assays.

### Luciferase assay

The ~150bp *Pdgfrb* promoter (bp-116 to +25, relative to the transcription start site) [52] was PCR amplified using an upstream primer (5’-ATCGACGCGTCCACCCTCCCTGCTCCACCGC) and a downstream primer (5’-AGCTCTCGAGGAGCTCACACCACTGTGGGCTTTCTCTG), digested with MluI and XhoI, and cloned into the pGL3-basic luciferase reporter vector (Promega). The mutant constructs, in which the two SP1 binding sites were disrupted individually or in combination, were acquired through PCR-directed mutagenesis using the wild type reporter construct as the parental plasmid. All constructs were confirmed with DNA sequencing. tsA58-AVM cells were electroporated with reporter constructs and then treated with different concentrations of TGFβ1 for 48 hours, at which point luciferase activity was measured using the Luciferase Assay kit (Promega). A plasmid expressing a lacZ reporter under the control of a constitutively active promoter was co-transfected for normalization of transfection efficiency. The beta-gal activity was measured using the Beta-Glo Assay System (Promega). We examined 3–5 independent cell cultures for each condition. For each culture, luciferase activity was measured in triplicate.

### Chromatin immunoprecipitation followed by quantitative PCR (ChIP qPCR) analysis

ChIP analysis was performed using ChromaFlash High-Sensitivity ChIP Kit (Epigentek, #P-2027-24) following the manufacturer’s protocol. Briefly, 2x10^5^ tsA58-AVM cells grown under the restrictive condition were treated with 1.0% formaldehyde to cross-link proteins to DNA for 10 minutes at room temperature. Crosslinking reactions were stopped by adding glycine solution (final concentration 0.125M). Cells were spun down and lysed using the lysis buffer (0.2ml) provided in the kit. After incubating for 10 minutes on ice, samples were spun down to acquire chromatin pellet, which was resuspended in 0.2ml ChIP buffer (provided in the kit). Genomic DNA was sheared using Bioruptor XL sonicator (Diagenode) for 10 min at the M2 intensity level to acquire optimal DNA fragment size of ~300bp. 2ul of anti-SMAD2 antibody was added to 50ul antibody buffer (provide in the kit) to produce antibody solution, which was then added to assay-strip wells. After 1 hour incubation at room temperature, DNA-protein samples were then added to the wells that were pre-incubated with the anti-SMAD2 antibody. Precipitation of the DNA-protein complexes was performed overnight at 4°C followed by intensive washing. Samples were then digested with RNAase and Proteinase K. DNA was then purified using the column provided in the kit. DNA samples were analyzed by qPCR using Roche LightCycler480 Real Time PCR machine (UAB Heflin Genomic Core). The relative DNA amount was calculated using the ΔΔCt method. The primers to amplify the *Pdgfrb* promoter are 5’-TGAAAACAGACACACGCGTCC and 5’- CACACCACTGTGGGCTTTCTC. Pre-immune IgG was used as a negative control.

### Statistical tests

The array analysis was performed after quality control and normalization by Robust Multi-array Average (RMA) methods using Partek Genomics Suite (PGS). Group comparison as 1-way ANOVA was conducted assuming non-equal variances. False discovery rate (FDR) *p* value correction was performed for multi-hypothesis purpose. The gene list (*p*<0.05) was generated using pathway analysis including canonical pathway, gene-gene interaction network, and upstream regulator/inhibitor, organ-related functional changes, etc., by using Ingenuity Pathway Analysis (IPA, Redwood City, CA) software package. The heatmap was generated by hierarchical clustering methods after r-normalization of intensity. For all other studies, unpaired Student’s tests were performed to compare the control and experimental groups with *p<0*.*05* considered as statistical significance. Data were averaged from at least 3 independent biological replicates and were shown as mean ± standard error (SEM).

## Results and discussion

### Identification of TGFβ regulatory target genes in tsA58-AVM cells

**Fig 1:**
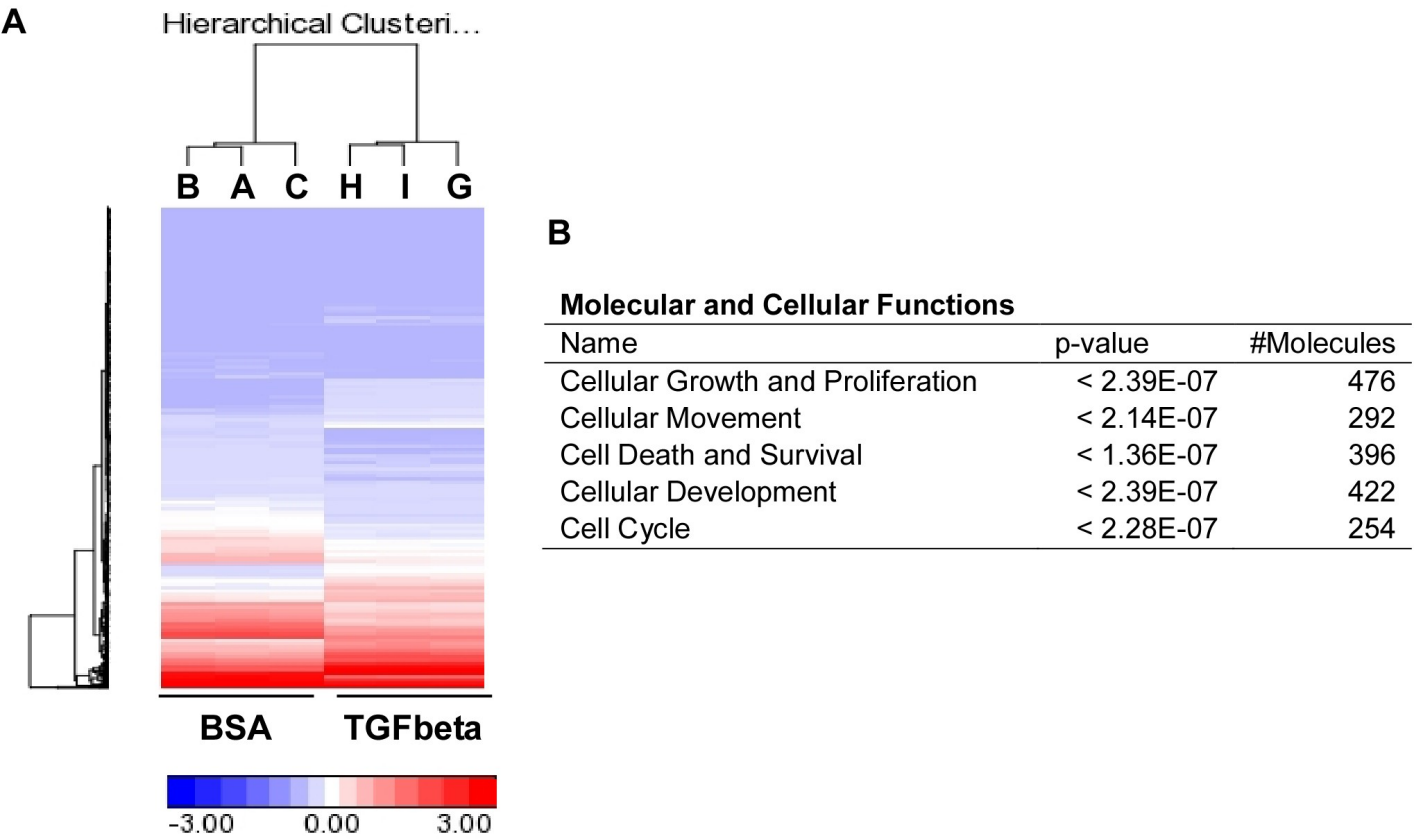
Identification of TGFβ regulatory targets in tsA58-AVM cells through microarray analysis. (A) tsA58-AVM cells were treated with 5ng/ml TGFβ1 or BSA for eight hours followed by microarray analysis. Data were averaged from three independent biological samples. This panel shows the heat map generated from microarray data. (B) Data from microarray analysis were analyzed with IPA to reveal the top five highest scoring hits in “Molecular and cellular functions”.

We recently established a conditional immortal AV cushion mesenchymal cell line, tsA58-AVM [39]. To identify regulatory target genes of TGFβ signaling, we treated tsA58-AVM cells, under the restrictive condition (37°C, without γ-interferon), with 5ng/ml of TGFβ1 or BSA followed by microarray analysis. The results in S1 Table and Fig 1A shows that expression of ~1,350 genes was significantly altered (False Discovery Rate *P<0*.*01*) between TGFβ-treated and control groups. Many known TGFβ downstream targets, such as *Jag1*, *Smad7*, *Snai1*, and Runx1, were identified. We next analyzed the data using the Ingenuity Pathway Analysis (IPA) software to reveal the biological functions that are mostly affected by TGFβ signaling. The top five highest scoring hits in “Molecular and Cellular Functions” are shown in Fig 1B. TGFβ1 treatment altered many genes involved in cellular growth, proliferation, death, survival and movement. This result supports the complex roles of TGFβ signaling in regulating AV cushion development and demonstrate the effectiveness of using tsA58-AVM cells to study the molecular/cellular mechanisms regulating cushion mesenchymal cells. This is particularly important given the limited quantity of cushion mesenchymal cells versus cardiomyocytes found in embryonic hearts. Moreover, a major advantage of using tsA58-AVM cells is that they resemble primary cells under the restrictive condition [39].

### PDGF-BB is the top upstream regulator of TGFβ activated genes in tsA58-AVM cells from “Upstream Regulator” analysis

**Fig 2:**
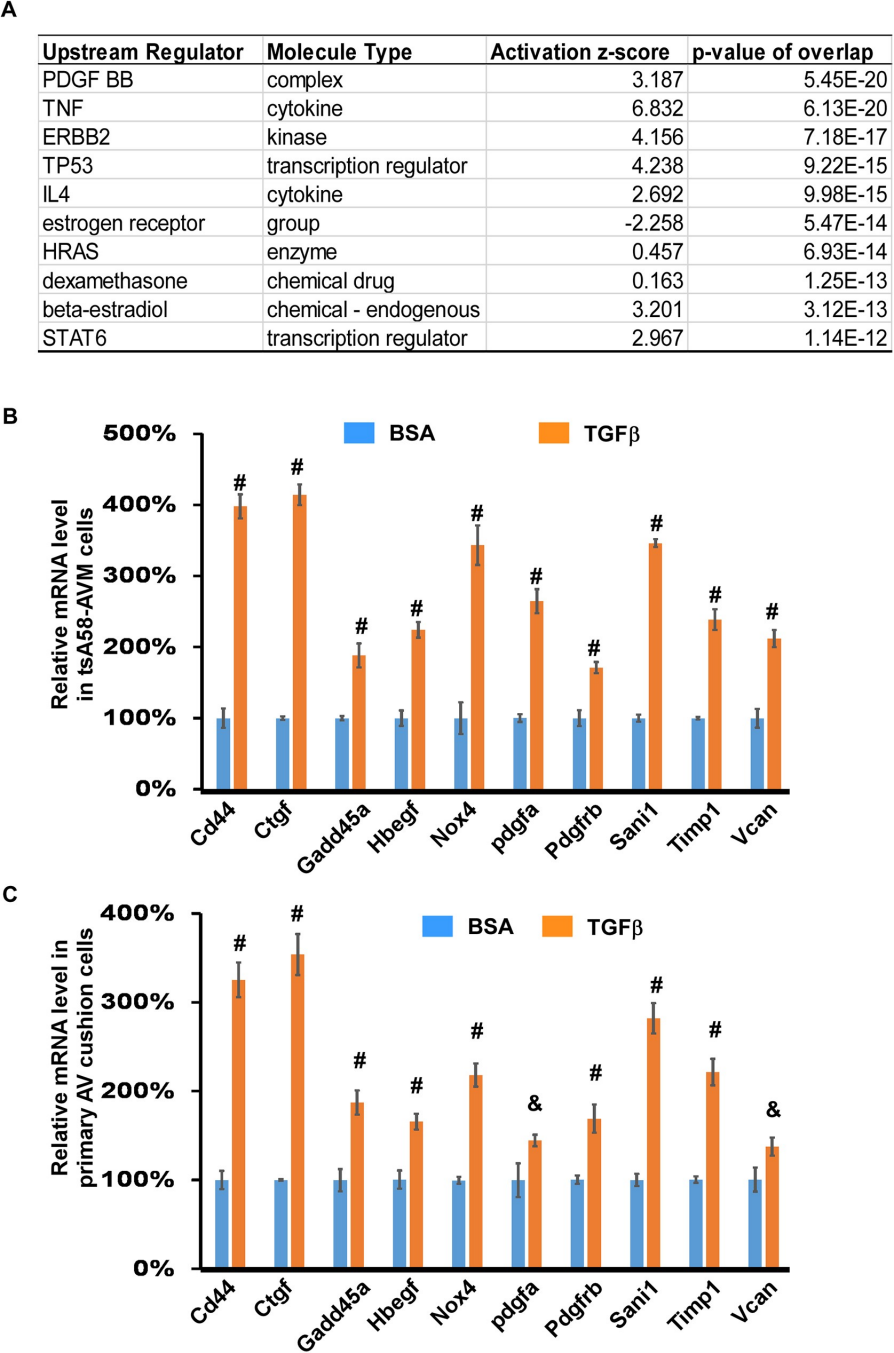
PDGF-BB is predicted to be the upstream regulator of TGFβ target genes. (A). The top 10 hits of the upstream regulators (excluding TGFβ ligands) of TGFβ regulatory targets revealed by bioinformatic analysis using the IPA software. (B) tsA58-AVM cells were treated with 5ng/ml TGFβ1 or BSA for eight hours followed by qRT-PCR analysis. The level of signals in cells treated with BSA was set at 100%. (C) The primary AV cushion cells were treated with TGFβ1 or BSA for six hours followed by qRT-PCR analysis. In panels B and C, data were averaged from three to five independent cultures with error bars indicating standard error (SEM). #: *p*<0.01, &: *p*<0.05, Student’s t test.

TGFβ signaling can both activate and inhibit gene transcription [16, 17], as observed in our microarray analysis (Fig 1, S2 Table). In this study, we primarily focused on the genes that are activated by the TGFβ1 ligand. We used the IPA software to perform “Upstream Regulator” analysis, which is used to identify the potential upstream regulators that are connected, directly or indirectly, to dataset genes [53]. The top 10 potential upstream regulators are shown in Fig 2A. PDGF-BB is the highest scoring excluding TGFB1, with a *p*-value of overlap as 5.45E-20. A total of 37 genes, which are activated by TGFβ signaling in tsA568-AVM cells, are predicted to be activated, directly or indirectly, by PDGF-BB (S3 Table). Based on the definition of “Upstream Regulator” analysis, upregulation of these genes by TGFβ1 treatment can potentially be explained as increased PDGF-BB signaling in these cells. Essential functions of PDGF-BB during atrioventricular valve development was demonstrated in a previous gene inactivation study [42]. We chose to perform qRT-PCR analysis on 10 genes from the 37 to test whether they are truly upregulated by TGFβ signaling. These selected genes are known to be involved in heart valve development and/or hemostasis. The result in Fig 2B shows that expression of all these genes was significantly increased by TGFβ treatment in both tsA58-AVM cells and primary AV cushion cells, validating the result acquired from our microarray analysis. The bioinformatic analysis result suggests a potentially functional interaction between TGFβ and PDGF signaling pathways during heart valve development. In future studies we will use mouse genetic approaches to test their interaction.

### The *Pdgfrb* promoter is responsive to TGFβ stimulation

**Fig 3:**
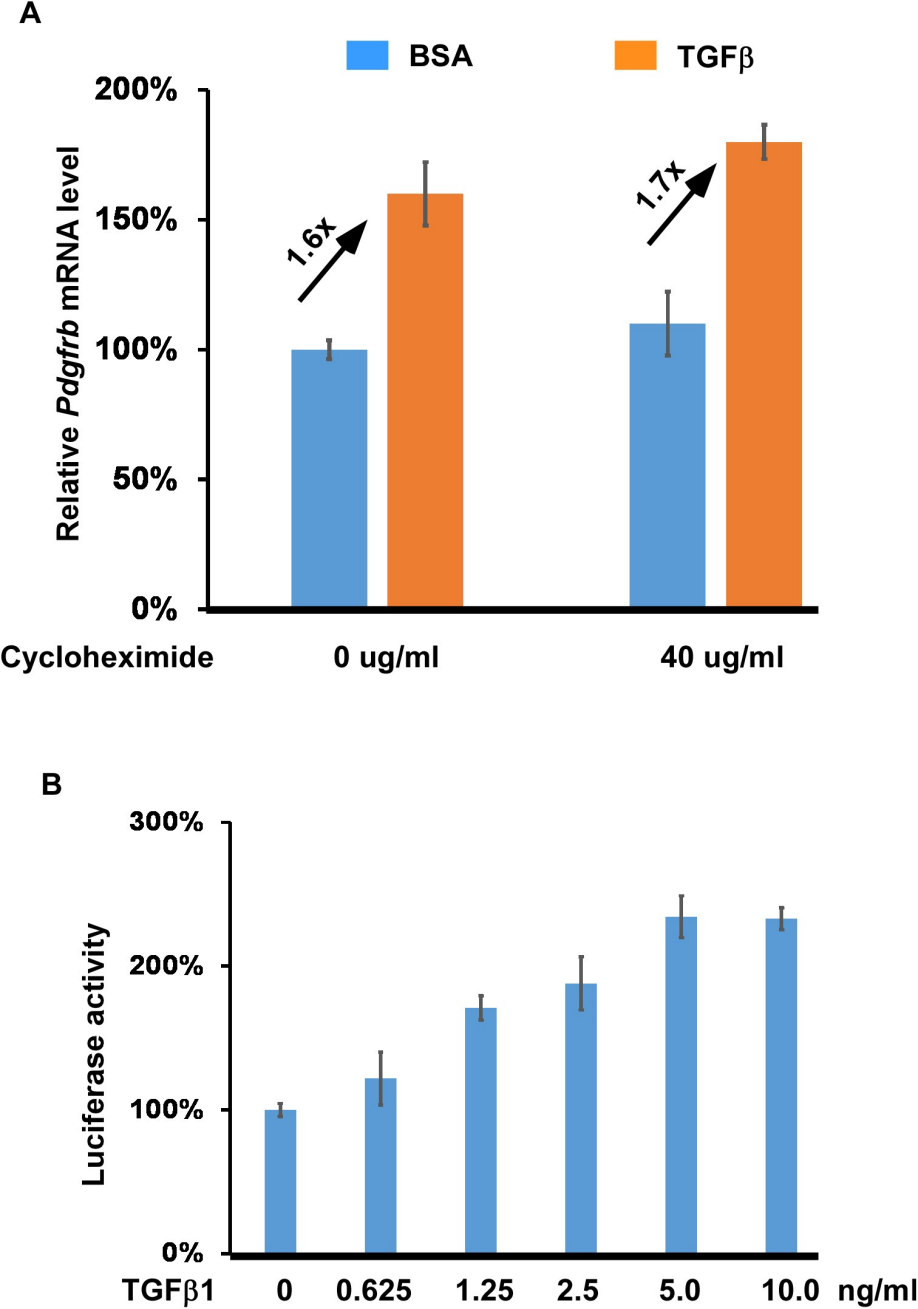
TGFβ signaling enhances *Pdgfrb* expression in tsA58-AVM cells. (A) tsA58-AVM cells were treated with TGFβ1 (5ng/ml) and/or cycloheximide (40ug/ml) for eight hours followed by qRT-PCR analysis to detect expression of *Pdgfrb*. The level of *Pdgfrb* expression in cells without TGFβ and cycloheximide treatment was set at 100%. (B) The *Pdgfrb* promoter region (bp-116 to +25, relative the transcription start site) was inserted into the pGL3-basic luciferase reporter vector (Promega). The reporter construct was then transfected into tsA58-AVM cells, which were then treated with different concentrations of TGFβ1 for 48 hours. Luciferase activity was measured. The level of luciferase activity observed in cells without TGFβ1 treatment was set at 100%. Data were averaged from three to five independent cultures with error bars indicating SEM.

We noticed that *Pdgfrb* is among the list of 37 genes in S3 Table. Our qRT-PCR analysis confirmed that expression of *Pdgfrb* is increased by ~60% upon TGFβ stimulation (Fig 2B). This gene encodes a cell-surface tyrosine kinase that can be activated by PDGF-BB [41], and therefore upregulated expression of this gene will lead to increased PDGF-BB signaling activity in tsA58-AVM cells. It was previously shown that overexpression of TRF1 increased expression of *PDGFRB* to ~1.6-fold over the control level and *PDGFRB* is a direct target of TRF1 in mediating its angiogenic activity [54]. This result suggests that the moderate increase in *Pdgfrb* expression may sufficiently lead to biological effects. As an initial step to determine whether *Pdgfrb* is a directly regulated by TGFβ signaling, we showed that upregulated expression of *Pdgfrb* by TGFβ1 does not rely on de novo protein synthesis (Fig 3A). Expression of *Pdgfrb* in cells treated with cycloheximide was still enhanced by TGFβ stimulation. Our next step was to use reporter analysis to show that the ~150bp promoter region of *Pdgfrb* [52] is responsive to TGFβ stimulation in a dose-dependent fashion in tsA58-AVM cells (Fig 3B). In addition to *Pdgfrb*, expression of several other genes involved in PDGF signaling, including *Pdgfa* and *Pdgfb*, were also upregulated by TGFβ stimulation (S2 Table). Upregulation of the two genes likely also contributes to the increased PDGF signaling in TGFβ treated cells. How TGFβ/SMAD signaling regulates expression of *Pdgfb* and *Pdgfa* will be further addressed in future studies.

### The SP1 sites within the *Pdgfrb* promoter are required for its responsiveness to TGFβ stimulation

**Fig 4:**
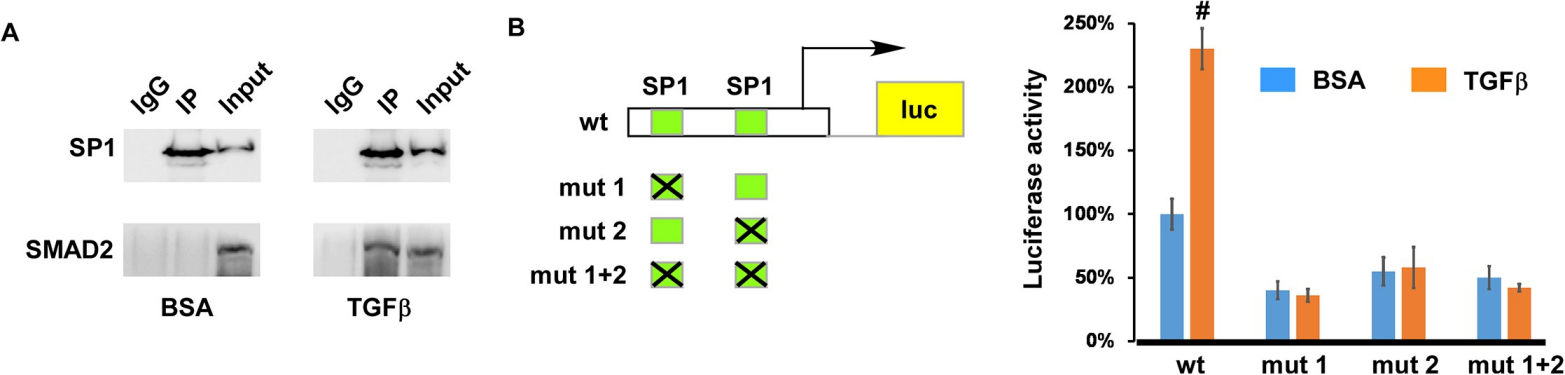
The SP1 sites are required for the *Pdgfrb* promoter to response to TGFβ stimulation. (A) tsA58-AVM cells were treated with TGFβ1 (5ng/ml) or BSA for 0.5 hours followed by co-IP analysis using an anti-SP1 antibody. Pre-immune rabbit IgG was used as the control. IP’d samples and inputs were subjected to Western analysis using antibodies to SP1 or SMAD2. (B) The promoter of *Pdgfrb* (bp-116 to +25, relative to the transcription start site) contains two SP1 binding sites. Various reporter constructs (wt, mut 1, mut 2 and mut 1+2) were electroporated into tsA58-AVM cells, which were then treated with or without TGFβ1 (5ng/ml) for 48 hours. Luciferase assay was then performed. The level of luciferase activity measured in cells harboring the wild type promoter construct without TGFβ1 treatment was set at 100%. Data were averaged from four to six independent cultures with error bars indicating SEM. #: *p*<0.01, Student’s t test.

We examined the *Pdgfrb* promoter using multiple cis-element prediction programs and failed to identify any potential SBEs within the promoter. It is well established that the ~150bp promoter contains two functional SP1 binding sites and they play essential roles in activating transcription of *Pdgfrb* [52]. The two SP1 target elements binds SP1 in a gel-mobility-shift assay and loss of the two sites eliminates the response of the *Pdgfrb* promoter to overexpression of SP1 [52]. Furthermore, SP1 co-operates with NF-Y to upregulate *Pdgfrb* transcription and the additive effect between the two factors relies on the presence of the two SP1 binding sites [52]. Considering that SP1 physically interacts with TGFβ R-SMADs in multiple cell types [55–57], we speculate that SP1 may co-operate with TGFβ-R SMADs to regulate *Pdgfrb* transcription. We decided to test whether SP1 recruits TGFβ R-SMADs to the *Pdgfrb* promoter to upregulate its expression upon TGFβ stimulation. In Fig 4A, we performed co-IP assays and show that SMAD2 is co-purified with SP1 in tsA58-AVM cells. Interaction between SMAD2 and SP1 relies on TGFβ treatment; this is expected as only when TGFβ signaling is activated, can SMAD2 be phosphorylated and transferred into the nucleus. In Fig 4B, we generated three mutant reporter constructs using the wild type construct as the parental plasmid. The two SP1 binding sites were mutated individually or in combination. Our reporter analysis showed that disruption of each single SP1 site or both of them simultaneously significantly reduced the basal activity of the *Pdgfrb* promoter. Furthermore, mutant reporters were unable to respond to TGFβ treatment. The results shown in Fig 4 collectively support the idea that TGFβ R-SMADs act through the SP1 sites to upregulate the activity of the *Pdgfrb* promoter.

### SMAD2 is associated with the *Pdgfrb* promoter and this association relies on SP1

**Fig 5:**
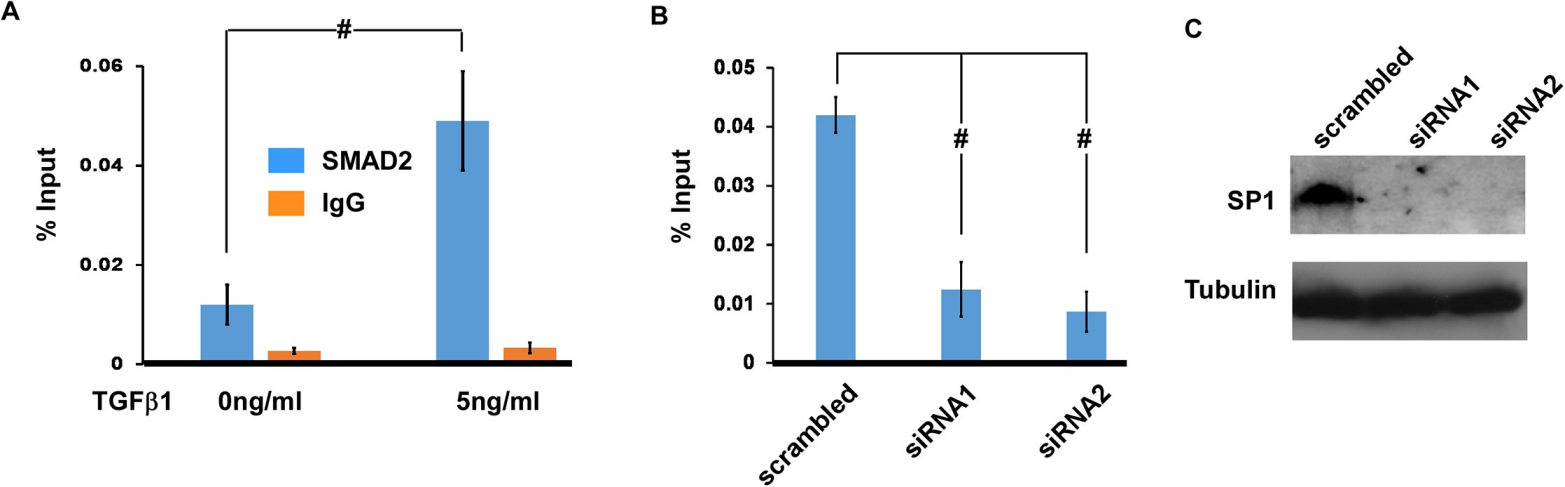
Association of the *Pdgfrb* promoter with SMAD2. (A) tsA58-AVM cells were treated with or without TGFβ1 (5ng/ml) for 1 hour followed by ChIP analysis using an anti-SMAD2 antibody. Pre-immune rabbit IgG was used as the control. ChIP’d samples were subjected to qPCR analysis using primers that corresponded to the *Pdgfrb* promoter. Data were normalized against input DNA. (B) tsA58-AVM cells were electroporated with DsiRNAs against *Sp1* or a scrambled control. Cells were then treated with TGFβ1 (5ng/ml) for 1 hour followed by ChIP-qPCR analysis to examine the association between SMAD2 and the *Pdgfrb* promoter. (C) Western analysis was performed to confirm that the two siRNAs can efficiently knock down expression of SP1. Data in panels A and B were averaged from three independent cultures with error bars indicating SEM. #: *p*<0.01, Student’s t test.

To directly test whether *Pdgfrb* is a direct regulatory target of TGFβ/SMAD2, we performed ChIP-qPCR analysis using an anti-SMAD2 antibody. As shown in Fig 5A, TGFβ stimulation significantly increased association of SMAD2 with the *Pdgfrb* promoter. Furthermore, we showed that knocking down expression of SP1 with two independent DsiRNAs significantly reduced association between SMAD2 and the *Pdgfrb* promoter. The efficiency of the two independent siRNAs to knock down SP1 expression was confirmed by Western analysis, shown in Fig 5C.

The interaction between TGFβ and PDGF signaling pathways in different cell types has been well documented in the literature (*e*.*g*. [46–48]). Our current study provides multiple lines of evidence showing for the first time, that TGFβ/SMAD2 can directly upregulate expression of *Pdgfrb*. We showed that upregulation of *Pdgfrb* mRNA expression by TGFβ does not require de novo protein synthesis. Subsequent reporter, co-IP and ChIP studies suggest that SMAD2 is recruited to the *Pdgfrb* promoter through interaction with SP1. Considering the established function of PDGF signaling in heart valve development [42], we speculate that PDGFRB acts as a critical downstream target of TGFβ signaling during valvulogenesis. Our study thus reveals a novel mechanism underlying the interaction between TGFβ and PDGF signaling pathways. Whether such a mechanism also exists in other cell types and/or during other biological processes warrants further investigation.

## Supporting information

S1 Table Primers used in this study. (DOCX)

S2 Table The list of genes whose expression is significantly altered by addition of TGFβ1. (XLS)

S3 Table The list of TGFβ1-activated genes whose expression is predicted to be activated by the PDGF-BB signaling. (XLSX)

## References

[1] Armstrong EJ, Bischoff J. Heart valve development: endothelial cell signaling and differentiation. Circ Res. 2004;95(5):459–70. PubMed Central PMCID: PMCPMC2810618. doi: 10.1161/01.RES.0000141146.95728.da 15345668 10.1161/01.RES.0000141146.95728.da PMC2810618

[2] Barnett JV, Desgrosellier JS. Early events in valvulogenesis: a signaling perspective. Birth Defects Res C Embryo Today. 2003;69(1):58–72. doi: 10.1002/bdrc.10006 12768658 10.1002/bdrc.10006

[3] Schroeder JA, Jackson LF, Lee DC, Camenisch TD. Form and function of developing heart valves: coordination by extracellular matrix and growth factor signaling. J Mol Med. 2003;81(7):392–403. doi: 10.1007/s00109-003-0456-5 12827270 10.1007/s00109-003-0456-5

[4] Person AD, Klewer SE, Runyan RB. Cell biology of cardiac cushion development. Int Rev Cytol. 2005;243:287–335. doi: 10.1016/S0074-7696(05)43005-3 15797462 10.1016/S0074-7696(05)43005-3

[5] Butcher JT, Markwald RR. Valvulogenesis: the moving target. Philos Trans R Soc Lond B Biol Sci. 2007;362(1484):1489–503. PubMed Central PMCID: PMCPMC2440410. doi: 10.1098/rstb.2007.2130 17569640 10.1098/rstb.2007.2130 PMC2440410

[6] de la Cruz MV, Markwald RR, Krug EL, Rumenoff L, Sanchez Gomez C, Sadowinski S, et al Living morphogenesis of the ventricles and congenital pathology of their component parts. Cardiol Young. 2001;11(6):588–600. 11813909 10.1017/s1047951101000932

[7] Gittenberger-de Groot AC, Bartelings MM, Deruiter MC, Poelmann RE. Basics of cardiac development for the understanding of congenital heart malformations. Pediatr Res. 2005;57(2):169–76. doi: 10.1203/01.PDR.0000148710.69159.61 15611355 10.1203/01.PDR.0000148710.69159.61

[8] Wagner M, Siddiqui MA. Signal transduction in early heart development (II): ventricular chamber specification, trabeculation, and heart valve formation. Exp Biol Med (Maywood). 2007;232(7):866–80. 17609502

[9] Lin CJ, Lin CY, Chen CH, Zhou B, Chang CP. Partitioning the heart: mechanisms of cardiac septation and valve development. Development. 2012;139(18):3277–99. Epub 2012/08/23. PubMed Central PMCID: PMCPMC3424040. doi: 10.1242/dev.063495 22912411 10.1242/dev.063495 PMC3424040

[10] Hinton RB, Yutzey KE. Heart valve structure and function in development and disease. Annu Rev Physiol. 2011;73:29–46. Epub 2010/09/03. PubMed Central PMCID: PMCPMC4209403. doi: 10.1146/annurev-physiol-012110-142145 20809794 10.1146/annurev-physiol-012110-142145 PMC4209403

[11] Markwald RR, Norris RA, Moreno-Rodriguez R, Levine RA. Developmental basis of adult cardiovascular diseases: valvular heart diseases. Ann N Y Acad Sci. 2010;1188:177–83. PubMed Central PMCID: PMCPMC3371607. doi: 10.1111/j.1749-6632.2009.05098.x 20201901 10.1111/j.1749-6632.2009.05098.x PMC3371607

[12] Calkoen EE, Hazekamp MG, Blom NA, Elders BB, Gittenberger-de Groot AC, Haak MC, et al Atrioventricular septal defect: From embryonic development to long-term follow-up. International journal of cardiology. 2016;202:784–95. doi: 10.1016/j.ijcard.2015.09.081 26476030 10.1016/j.ijcard.2015.09.081

[13] Shi Y, Massague J. Mechanisms of TGF-beta signaling from cell membrane to the nucleus. Cell. 2003;113(6):685–700. 12809600 10.1016/s0092-8674(03)00432-x

[14] de Caestecker M. The transforming growth factor-beta superfamily of receptors. Cytokine Growth Factor Rev. 2004;15(1):1–11. 14746809 10.1016/j.cytogfr.2003.10.004

[15] ten Dijke P, Hill CS. New insights into TGF-beta-Smad signalling. Trends Biochem Sci. 2004;29(5):265–73. doi: 10.1016/j.tibs.2004.03.008 15130563 10.1016/j.tibs.2004.03.008

[16] Hata A, Chen YG. TGF-beta Signaling from Receptors to Smads. Cold Spring Harbor perspectives in biology. 2016;8(9). 10.1101/cshperspect.a022061 PMC5008074 27449815

[17] Macias MJ, Martin-Malpartida P, Massague J. Structural determinants of Smad function in TGF-beta signaling. Trends Biochem Sci. 2015;40(6):296–308. PubMed Central PMCID: PMCPMC4485443. doi: 10.1016/j.tibs.2015.03.012 25935112 10.1016/j.tibs.2015.03.012 PMC4485443

[18] Morikawa M, Koinuma D, Miyazono K, Heldin CH. Genome-wide mechanisms of Smad binding. Oncogene. 2013;32(13):1609–15. PubMed Central PMCID: PMCPMC3615190. doi: 10.1038/onc.2012.191 22614010 10.1038/onc.2012.191 PMC3615190

[19] Massague J, Seoane J, Wotton D. Smad transcription factors. Genes Dev. 2005;19(23):2783–810. doi: 10.1101/gad.1350705 16322555 10.1101/gad.1350705

[20] Feng XH, Derynck R. Specificity and versatility in tgf-beta signaling through Smads. Annu Rev Cell Dev Biol. 2005;21:659–93. doi: 10.1146/annurev.cellbio.21.022404.142018 16212511 10.1146/annurev.cellbio.21.022404.142018

[21] Datto M, Wang XF. The Smads: transcriptional regulation and mouse models. Cytokine Growth Factor Rev. 2000;11(1–2):37–48. 10708951 10.1016/s1359-6101(99)00027-1

[22] Potts JD, Runyan RB. Epithelial-mesenchymal cell transformation in the embryonic heart can be mediated, in part, by transforming growth factor beta. Dev Biol. 1989;134(2):392–401. 2744239 10.1016/0012-1606(89)90111-5

[23] Nakajima Y, Yamagishi T, Hokari S, Nakamura H. Mechanisms involved in valvuloseptal endocardial cushion formation in early cardiogenesis: roles of transforming growth factor (TGF)-beta and bone morphogenetic protein (BMP). Anat Rec. 2000;258(2):119–27. 10645959 10.1002/(SICI)1097-0185(20000201)258:2<119::AID-AR1>3.0.CO;2-U

[24] Ramsdell AF, Markwald RR. Induction of endocardial cushion tissue in the avian heart is regulated, in part, by TGFbeta-3-mediated autocrine signaling. Dev Biol. 1997;188(1):64–74. doi: 10.1006/dbio.1997.8637 9245512 10.1006/dbio.1997.8637

[25] Brown CB, Boyer AS, Runyan RB, Barnett JV. Antibodies to the Type II TGFbeta receptor block cell activation and migration during atrioventricular cushion transformation in the heart. Dev Biol. 1996;174(2):248–57. doi: 10.1006/dbio.1996.0070 8631497 10.1006/dbio.1996.0070

[26] Boyer AS, Ayerinskas II, Vincent EB, McKinney LA, Weeks DL, Runyan RB. TGFbeta2 and TGFbeta3 have separate and sequential activities during epithelial-mesenchymal cell transformation in the embryonic heart. Dev Biol. 1999;208(2):530–45. doi: 10.1006/dbio.1999.9211 10191064 10.1006/dbio.1999.9211

[27] Brown CB, Boyer AS, Runyan RB, Barnett JV. Requirement of type III TGF-beta receptor for endocardial cell transformation in the heart. Science. 1999;283(5410):2080–2. 10092230 10.1126/science.283.5410.2080

[28] Camenisch TD, Molin DG, Person A, Runyan RB, Gittenberger-de Groot AC, McDonald JA, et al Temporal and distinct TGFbeta ligand requirements during mouse and avian endocardial cushion morphogenesis. Dev Biol. 2002;248(1):170–81. 12142029 10.1006/dbio.2002.0731

[29] Sanford LP, Ormsby I, Gittenberger-de Groot AC, Sariola H, Friedman R, Boivin GP, et al TGFbeta2 knockout mice have multiple developmental defects that are non-overlapping with other TGFbeta knockout phenotypes. Development. 1997;124(13):2659–70. 9217007 10.1242/dev.124.13.2659 PMC3850286

[30] Bartram U, Molin DG, Wisse LJ, Mohamad A, Sanford LP, Doetschman T, et al Double-outlet right ventricle and overriding tricuspid valve reflect disturbances of looping, myocardialization, endocardial cushion differentiation, and apoptosis in TGF-beta(2)-knockout mice. Circulation. 2001;103(22):2745–52. 11390347 10.1161/01.cir.103.22.2745

[31] Azhar M, Brown K, Gard C, Chen H, Rajan S, Elliott DA, et al Transforming growth factor Beta2 is required for valve remodeling during heart development. Dev Dyn. 2011;240(9):2127–41. PubMed Central PMCID: PMCPMC3337781. doi: 10.1002/dvdy.22702 21780244 10.1002/dvdy.22702 PMC3337781

[32] Dickson MC, Martin JS, Cousins FM, Kulkarni AB, Karlsson S, Akhurst RJ. Defective haematopoiesis and vasculogenesis in transforming growth factor-beta 1 knock out mice. Development. 1995;121(6):1845–54. 7600998 10.1242/dev.121.6.1845

[33] Kaartinen V, Voncken JW, Shuler C, Warburton D, Bu D, Heisterkamp N, et al Abnormal lung development and cleft palate in mice lacking TGF-beta 3 indicates defects of epithelial-mesenchymal interaction. Nat Genet. 1995;11(4):415–21. doi: 10.1038/ng1295-415 7493022 10.1038/ng1295-415

[34] Jiao K, Langworthy M, Batts L, Brown CB, Moses HL, Baldwin HS. Tgfbeta signaling is required for atrioventricular cushion mesenchyme remodeling during in vivo cardiac development. Development. 2006;133(22):4585–93. doi: 10.1242/dev.02597 17050629 10.1242/dev.02597

[35] Wang J, Sridurongrit S, Dudas M, Thomas P, Nagy A, Schneider MD, et al Atrioventricular cushion transformation is mediated by ALK2 in the developing mouse heart. Dev Biol. 2005;286(1):299–310. doi: 10.1016/j.ydbio.2005.07.035 16140292 10.1016/j.ydbio.2005.07.035 PMC1361261

[36] Sridurongrit S, Larsson J, Schwartz R, Ruiz-Lozano P, Kaartinen V. Signaling via the Tgf-beta type I receptor Alk5 in heart development. Dev Biol. 2008;322(1):208–18. Epub 2008/08/23. PubMed Central PMCID: PMC2677203. doi: 10.1016/j.ydbio.2008.07.038 18718461 10.1016/j.ydbio.2008.07.038 PMC2677203

[37] Song L, Zhao M, Wu B, Zhou B, Wang Q, Jiao K. Cell autonomous requirement of endocardial Smad4 during atrioventricular cushion development in mouse embryos. Dev Dyn. 2011;240(1):211–20. doi: 10.1002/dvdy.22493 21089072 10.1002/dvdy.22493 PMC3020975

[38] Moskowitz IP, Wang J, Peterson MA, Pu WT, Mackinnon AC, Oxburgh L, et al Transcription factor genes Smad4 and Gata4 cooperatively regulate cardiac valve development. [corrected]. Proc Natl Acad Sci U S A. 2011;108(10):4006–11. PubMed Central PMCID: PMCPMC3053967. doi: 10.1073/pnas.1019025108 21330551 10.1073/pnas.1019025108 PMC3053967

[39] Peng Y, Song L, Li D, Kesterson R, Wang J, Wang L, et al Sema6D acts downstream of bone morphogenetic protein signalling to promote atrioventricular cushion development in mice. Cardiovasc Res. 2016. 10.1093/cvr/cvw200 PMC5901116 28172500

[40] Paulsson J, Ehnman M, Ostman A. PDGF receptors in tumor biology: prognostic and predictive potential. Future Oncol. 2014;10(9):1695–708. doi: 10.2217/fon.14.83 25145436 10.2217/fon.14.83

[41] Demoulin JB, Essaghir A. PDGF receptor signaling networks in normal and cancer cells. Cytokine Growth Factor Rev. 2014;25(3):273–83. doi: 10.1016/j.cytogfr.2014.03.003 24703957 10.1016/j.cytogfr.2014.03.003

[42] Van den Akker NM, Winkel LC, Nisancioglu MH, Maas S, Wisse LJ, Armulik A, et al PDGF-B signaling is important for murine cardiac development: its role in developing atrioventricular valves, coronaries, and cardiac innervation. Dev Dyn. 2008;237(2):494–503. doi: 10.1002/dvdy.21436 18213589 10.1002/dvdy.21436

[43] Leveen P, Pekny M, Gebre-Medhin S, Swolin B, Larsson E, Betsholtz C. Mice deficient for PDGF B show renal, cardiovascular, and hematological abnormalities. Genes Dev. 1994;8(16):1875–87. 7958863 10.1101/gad.8.16.1875

[44] Hellstrom M, Kalen M, Lindahl P, Abramsson A, Betsholtz C. Role of PDGF-B and PDGFR-beta in recruitment of vascular smooth muscle cells and pericytes during embryonic blood vessel formation in the mouse. Development. 1999;126(14):3047–55. 10375497 10.1242/dev.126.14.3047

[45] Bjarnegard M, Enge M, Norlin J, Gustafsdottir S, Fredriksson S, Abramsson A, et al Endothelium-specific ablation of PDGFB leads to pericyte loss and glomerular, cardiac and placental abnormalities. Development. 2004;131(8):1847–57. doi: 10.1242/dev.01080 15084468 10.1242/dev.01080

[46] Lee JI, Wright JH, Johnson MM, Bauer RL, Sorg K, Yuen S, et al Role of Smad3 in platelet-derived growth factor-C-induced liver fibrosis. Am J Physiol Cell Physiol. 2016;310(6):C436–45. PubMed Central PMCID: PMCPMC4796282. doi: 10.1152/ajpcell.00423.2014 26632601 10.1152/ajpcell.00423.2014 PMC4796282

[47] Liu C, Li J, Xiang X, Guo L, Tu K, Liu Q, et al PDGF receptor-alpha promotes TGF-beta signaling in hepatic stellate cells via transcriptional and posttranscriptional regulation of TGF-beta receptors. Am J Physiol Gastrointest Liver Physiol. 2014;307(7):G749–59. PubMed Central PMCID: PMCPMC4187064. doi: 10.1152/ajpgi.00138.2014 25169976 10.1152/ajpgi.00138.2014 PMC4187064

[48] Martin-Garrido A, Williams HC, Lee M, Seidel-Rogol B, Ci X, Dong JT, et al Transforming growth factor beta inhibits platelet derived growth factor-induced vascular smooth muscle cell proliferation via Akt-independent, Smad-mediated cyclin D1 downregulation. PLoS One. 2013;8(11):e79657 PubMed Central PMCID: PMCPMC3827379. doi: 10.1371/journal.pone.0079657 24236150 10.1371/journal.pone.0079657 PMC3827379

[49] Peng Y, Song L, Zhao M, Harmelink C, Debenedittis P, Cui X, et al Critical roles of miRNA-mediated regulation of TGFbeta signalling during mouse cardiogenesis. Cardiovasc Res. 2014;103(2):258–67. PubMed Central PMCID: PMCPMC4110444. doi: 10.1093/cvr/cvu126 24835278 10.1093/cvr/cvu126 PMC4110444

[50] Peng Y, Song L, Li D, Kesterson R, Wang J, Wang L, et al Sema6D acts downstream of bone morphogenetic protein signalling to promote atrioventricular cushion development in mice. Cardiovasc Res. 2016;112(2):532–42. doi: 10.1093/cvr/cvw200 28172500 10.1093/cvr/cvw200 PMC5901116

[51] Liu Y, Harmelink C, Peng Y, Chen Y, Wang Q, Jiao K. CHD7 interacts with BMP R-SMADs to epigenetically regulate cardiogenesis in mice. Hum Mol Genet. 2014;23(8):2145–56. Epub 2013/12/03. PubMed Central PMCID: PMCPMC3959819. doi: 10.1093/hmg/ddt610 24293546 10.1093/hmg/ddt610 PMC3959819

[52] Molander C, Hackzell A, Ohta M, Izumi H, Funa K. Sp1 is a key regulator of the PDGF beta-receptor transcription. Molecular biology reports. 2001;28(4):223–33. 12153142 10.1023/a:1015701232589

[53] Kramer A, Green J, Pollard J Jr., Tugendreich S. Causal analysis approaches in Ingenuity Pathway Analysis. Bioinformatics. 2014;30(4):523–30. PubMed Central PMCID: PMCPMC3928520. doi: 10.1093/bioinformatics/btt703 24336805 10.1093/bioinformatics/btt703 PMC3928520

[54] El Mai M, Wagner KD, Michiels JF, Ambrosetti D, Borderie A, Destree S, et al The Telomeric Protein TRF2 Regulates Angiogenesis by Binding and Activating the PDGFRbeta Promoter. Cell Rep. 2014;9(3):1047–60. doi: 10.1016/j.celrep.2014.09.038 25437559 10.1016/j.celrep.2014.09.038

[55] Pardali K, Kurisaki A, Moren A, ten Dijke P, Kardassis D, Moustakas A. Role of Smad proteins and transcription factor Sp1 in p21(Waf1/Cip1) regulation by transforming growth factor-beta. J Biol Chem. 2000;275(38):29244–56. doi: 10.1074/jbc.M909467199 10878024 10.1074/jbc.M909467199

[56] Feng XH, Lin X, Derynck R. Smad2, Smad3 and Smad4 cooperate with Sp1 to induce p15(Ink4B) transcription in response to TGF-beta. EMBO J. 2000;19(19):5178–93. PubMed Central PMCID: PMCPMC302105. doi: 10.1093/emboj/19.19.5178 11013220 10.1093/emboj/19.19.5178 PMC302105

[57] Jungert K, Buck A, Buchholz M, Wagner M, Adler G, Gress TM, et al Smad-Sp1 complexes mediate TGFbeta-induced early transcription of oncogenic Smad7 in pancreatic cancer cells. Carcinogenesis. 2006;27(12):2392–401. doi: 10.1093/carcin/bgl078 16714330 10.1093/carcin/bgl078

