# Supplementary material for: Pdgfrb is a direct regulatory target of TGFβ signaling in atrioventricular cushion mesenchymal cells
Source: PLoS One. 2017 Apr 20;12(4):e0175791. doi: 10.1371/journal.pone.0175791 (PMC5398542; doi:10.1371/journal.pone.0175791)
Supplement: S1 Table — (DOCX) [file pone.0175791.s001.docx]

**S1. Table. Primers used in this study**

| **Genes to be tested** | **Primer Sequences**  **(5’ to 3’)** | |
| --- | --- | --- |
| *Cd44* [1] | Forward  Backward | agcagcggctccaccatcgag  tcggatccatgagtcacagtg |
|  |  |  |
| *Ctgf* [2] | Forward  Backward | cttctgcgatttcggctcc  Tacaccgacccaccgaag |
| *Gadd45a* [3] | Forward  Backward | Ctgcctcctggtcacgaa  ttgcctctgctctcttcac |
| *Hbegf* | Forward  Backward | cagatacctgcaggagttcc  tcataacctcctctcctgtgg |
|  |  |  |
| *Nox4* | Forward  Backward | ggagtcactccatttgcatcg  tgactgaggtacagctggatg |
|  |  |  |
| *Pdgfa* [4] | Forward  Backward | ctcttggagatagactccgtagg  acttctcttcctgcgaatgg |
| *Pdgfrb* | Forward  Backward | gtgacagactacctctttgg  ctacatctcccagtgtctcc |
| *Snai1* [5] | Forward  Backward | CATCCTCGCTGGCATCTTCC  GAGAGCCAAGCAGGAACCAG |
|  |  |  |
| *Timp1* | Forward  Backward | ggtgtgcacagtgtttccctg  tccgtccacaaacagtgagtg |
|  |  |  |
| *Vcan* | Forward  Backward | TTGGCATTAGTGAAGAGTCCG  AACATAACTTGGGAGACAGAGAC |

**Referenes for S1. Table.**

1. Cohran V, Managlia E, Bradford EM, Goretsky T, Li T, Katzman RB, et al. Epithelial PIK3R1 (p85) and TP53 Regulate Survivin Expression during Adaptation to Ileocecal Resection. Am J Pathol. 2016;186(7):1837-46. doi: 10.1016/j.ajpath.2016.03.008. PubMed PMID: 27157990; PubMed Central PMCID: PMCPMC4929398.

2. Okada H, Kikuta T, Kobayashi T, Inoue T, Kanno Y, Takigawa M, et al. Connective tissue growth factor expressed in tubular epithelium plays a pivotal role in renal fibrogenesis. Journal of the American Society of Nephrology : JASN. 2005;16(1):133-43. doi: 10.1681/ASN.2004040339. PubMed PMID: 15574513.

3. Szele E, Gombos K, Kovacs A, Ember I. Feeding purified glycerol from biodiesel to CBA/CA mice: effects on Gadd45a and Nfkappab1 expressions. In Vivo. 2010;24(3):303-7. PubMed PMID: 20555003.

4. Tang Z, Arjunan P, Lee C, Li Y, Kumar A, Hou X, et al. Survival effect of PDGF-CC rescues neurons from apoptosis in both brain and retina by regulating GSK3beta phosphorylation. J Exp Med. 2010;207(4):867-80. doi: 10.1084/jem.20091704. PubMed PMID: 20231377; PubMed Central PMCID: PMCPMC2856029.

5. Bell CE, Watson AJ. SNAI1 and SNAI2 are asymmetrically expressed at the 2-cell stage and become segregated to the TE in the mouse blastocyst. PLoS One. 2009;4(12):e8530. doi: 10.1371/journal.pone.0008530. PubMed PMID: 20046880; PubMed Central PMCID: PMCPMC2796167.
